# Oxidative Stress, Induced by Sub-Lethal Doses of BDE 209, Promotes Energy Management and Cell Cycle Modulation in the Marine Fish Cell Line SAF-1

**DOI:** 10.3390/ijerph16030474

**Published:** 2019-02-06

**Authors:** Cristobal Espinosa Ruiz, Simona Manuguerra, Alberto Cuesta, Andrea Santulli, Concetta M. Messina

**Affiliations:** 1 Laboratory of Marine biochemistry and ecotoxicology, Department of Earth and Marine Science DISTEM, University of Palermo, Via Barlotta 4, 91100 Trapani, Italy; cristobal.espinosaruiz@unipa.it (C.E.R.); simona.manuguerra@unipa.it (S.M.); andrea.santulli@unipa.it (A.S.); 2 Fish Innate Immune System Group, Department of Cell Biology and Histology, Faculty of Biology, Campus Regional de Excelencia Internacional “Campus Mare Nostrum”, University of Murcia, 30100 Murcia, Spain; alcuesta@um.es; 3 Marine Biology Institute, Consorzio Universitario della Provincia di Trapani, Via Barlotta 4, 91100 Trapani, Italy

**Keywords:** PBDE-209, *Sparus aurata* fibroblast, oxidative stress, biomarkers

## Abstract

The effects of sub-lethal doses of polybrominated diphenyl ether (PBDE)-209 in terms of toxicity, oxidative stress, and biomarkers were evaluated in the *Sparus aurata* fibroblast cell line (SAF-1). Vitality and oxidative stress status were studied after incubation with PBDE for 72 h. Concomitantly, the quantification of proteins related to cell cycle and DNA repair (p53), cell proliferation (extracellular signal–regulated kinase 1 (ERK1)), energetic restriction (hypoxia-inducible factor 1 (HIF1)), and redox status (Nuclear factor erythroid 2–related factor 2 (NRF2)) was also determined after prolonged exposure (7–15 days) by immunoblotting. Our results demonstrated that rising concentrations of PBDEs exposure-induced oxidative stress, and that this event modulates different cell pathways related to cell cycle, cell signaling, and energetic balance in the long term, indicating the negative impact of sub-lethal dose exposure to cell homeostasis.

## 1. Introduction

Polybrominated diphenyl ethers (PBDEs) are a group of stable chemical compounds widely used in diverse polymers and plastics as flame retardants [1,2]. Among them, 2,2′,3,3′,4,4′,5,5′,6,6′-decabromodiphenyl ether (BDE-209) is the principal component found in commercial mixtures of PBDEs [3,4]. Regrettably, due to its properties, PBDEs accumulate in aquatic environments where they are ubiquitous, toxic, and persistent [5,6,7,8]. In addition, under certain environmental conditions it has been suggested that BDE-209 can be transformed into lower-brominated PBDEs congeners by physics and biological processes [9].

BDE-209 and its congeners have been demonstrated to produce negative effects on human health [10,11,12] and the health of laboratory/wild animals [13,14,15]. In fact, BDE-209 has been shown to produce oxidative stress and toxicity [16], affecting the thyroid endocrine system in fish and mammals [17,18,19,20], while PBDEs congeners are able to impair behavior, growth, reproductive, hepatic, and renal functions as well as immune and endocrine systems in fish [16,17,21,22,23,24,25,26].

It has been proposed that marine contaminants, contributing to the degradation of the nursery habitat and influencing the general welfare of fish have led to the decline of some wild marine fish populations [27] and to the alteration of the productivity of some marine fish stocks, due to the accumulation of pollutants [28,29,30]. For these reasons, the study of the effects and mechanisms determined by these toxicants in fish has attracted much attention. In this sense, the use of in vitro systems, represented by primary or permanent cell lines, or in vitro explants is considered more and more useful for studying the mechanisms of toxicology and represents a good alternative to in vivo trials with fish [31].

Different in vitro experiments have demonstrated that PBDEs have the ability to induce altered cell proliferation, as shown in the female reproductive system (human ovarian carcinoma cell line (OVCAR-3)) and normal ovarian (chinese hamster ovary cell line (CHO)) cells, mediated via phosphorylation of PKCα and ERK1/2 proteins [32], or induce the up-regulation of p53 and p23 in Neuro-2a cells [33]. In addition, toxicity, oxidative stress, and cell cycle arrest were described on spermatocytes from mice [34] and HepG2 cell lines treated with PBDEs [35]. In living organisms, PBDEs may be metabolized to more polar compounds—as shown experimentally in exposed mice and rats—that transform into hydroxylated, methoxylated, and/or conjugated metabolites (OH-, MeO- and GS-PBDEs, respectively) via phase I and phase II metabolic enzymes [36,37].

In the case of fish, molecular mechanisms by which the PBDEs affect viability and cell cycle are still unknown, although oxidative stress has been suggested as the principal process involved in cell aggression. On the other hand, it is reasonable to think that the fact there is no toxicant effect does not mean the compounds are not producing cell damage. In fact, exposure to sub-lethal doses could promote other adverse effects after long-term exposure. Current knowledge about the sub-lethal effects of BDE-209 in aquatic organisms is still insufficient [17]. Because of this, the evaluation of biomarkers that could detect the negative effects of sub-lethal concentrations of PBDEs may be of great interest. In addition, as far as we know, very few papers have evaluated the effect of PBDEs using marine fish cell lines [38,39]. With the aim to clarify some of these aspects, we evaluated the effect of PBDEs in the *Sparus aurata* fibroblast cell line (SAF-1) model system.

Vitality and oxidative stress status were studied after incubation with PBDEs for a short time (72 h). Additionally, the quantification of proteins related to cell cycle (p53, a protein involved on cell cycle arrest and DNA repair), cell proliferation (extracellular signal–regulated kinase 1 (ERK1), a kinase involved on cell proliferation through complex signaling pathways), energetic balance (hypoxia-inducible factor 1 (HIF1), a protein complex regulated by oxygen availability, able to modulate glycolytic enzymes and ATP production), and oxidative stress (Nuclear factor erythroid 2–related factor 2 (NRF2), considered as an oxidative stress sensor that activates the antioxidant and detoxifying response) were determined after a prolonged exposure (7–15 days).

## 2. Material and Methods

### 2.1. SAF-1 Cell Culture

The established cell line SAF-1 (ECACC n°00122301), from fibroblast of *Sparus aurata*, was seeded in 25 cm^2^ plastic tissue culture flasks (Nunc, Darmstadt, Germany) cultured in L-15 Leibowitz medium (Sigma, Haverhill, UK), supplemented with 10% fetal bovine serum (FBS, Sigma, UK), 2 mmol L^−1^ L-glutamine (Sigma, UK), 100 i.u. mL^−1^ penicillin (Sigma, UK), and 100 g L^−1^ streptomycin (Sigma, UK). Cells were grown at 25 °C in a humidified atmosphere (85% humidity).

Exponentially growing cells were detached from culture flasks by brief exposure to 0.25% of trypsin in PBS, pH 7.2–7.4, according to the standard trypsinization methods. The detached cells were collected by centrifugation (11,200× *g*, 5 min, 25 °C), and the cell vitality was determined by the trypan blue exclusion test.

### 2.2. Cytotoxicity Assay on SAF-1 Cell Line

Cytotoxicity assay was performed in five replicates. When SAF-1 cell lines were approximately 80% confluent, they were detached from flasks cultured with trypsin (as described before), and aliquots of 100 μL containing 10,000 cell well^−1^ were dispensed in 96-well tissue culture plates and incubated (24 h, 25 °C). This cell concentration was pre-determined in order to obtain satisfactory absorbance values in the cytotoxic assay, and to avoid cell over-growth. After that, the culture medium was replaced by 100 μL well^−1^ of the PBDEs to be tested at the appropriate dilution.

The PBDE standard (100% of purity) was provided by SPECTRA (Rome, Italy); stock solution of BDE-209 at a concentration of 25 mmol L^−1^ was prepared by dissolving the powder compounds in dimethyl-sulfoxide (DMSO). Tested concentrations of BDE-209 ranged from 0.25 to 2 µmol L^−1^ (0.25, 0.5, 0.75, 1, and 2). Cells were then incubated for 24, 48, and 72 h in three different plates at 25 °C. Control samples received the same volume of culture medium and DMSO (0.1%), although the absence of the effects by the vehicle is well known [40,41]. After 24, 48, and 72 h at 25 °C, their vitality was determined using the tetrazolium salt (3-(4,5-dimethylthiazol-2-yl)-2,5-diphenyltetrazolium bromide) (MTT) assay.

The MTT assay is based on the reduction of the yellow soluble MTT (Sigma-Aldrich, Saint Louis, USA) into a blue, insoluble formazan product by the mitochondrial succinate dehydrogenase [42,43]. After incubation with the PDEs, SAF-1 cells were washed with phosphate buffer saline solution (PBS) and 200 μL well^−1^ of MTT (1 g L^−1^) was added. After 4 h of incubation, cells were washed again and the formazan crystals solubilized with 100 μL well^−1^ of DMSO. Plates were shacked (5 min, 100 rpm) in dark conditions, and the absorbance at 570 nm and 690 nm was determined in a microplate reader (Opsys MR™ Microplate Reader, DYNEX TECHNOLOGIES INC., Chantilly, VA, USA). After the individuation of the sub-lethal concentrations, the next experiments were done in order to assess molecular markers related to different biochemical patterns.

### 2.3. Evaluation of Intracellular Reactive Oxygen Species (ROS)

SAF-1 cells were incubated with different concentrations of BDE-209, as described above. After 72 h of treatment, intracellular ROS were analyzed on cell seeded in 96 microplate, using the dichlorodihydrofluorescein-diacetate (DCF-DA) method [44] with some modifications [41]. DCF-DA is oxidized to dichlorodihydrofluorescein (DCF) by ROS. Each well was exposed to 10 µL of DCF-DA in HBSS (5 mg L^−1^), incubated for 5 min at 37 °C to allow the oxidation of the DCF-DA, and successively read on a spectrofluorometer (485 exc–530 em, Varian Cary Eclipse, Mulgrave, Victoria, Australia). The results have been expressed as relative fluorescence/µg of total proteins (rf µg tp^−1^).

#### 2.3.1. Evaluation of Molecular Markers by Immunoblotting

For the evaluation of molecular markers related to the different pathways of oxidative stress, cell cycle progression, apoptosis, and energy balance management, a long-term experiment lasting 15 days was carried out in flask, for each compound, at only one sub-lethal concentration (1 µmol L^−1^). This dose was chosen as it corresponds to the range in which both the cell vitality and the ROS production changed, and could therefore return us information on the molecular patterns activated by the PBDE in the long term.

SAF-1 cells (40,000 cells cm^−2^) were incubated in a 25 cm^2^ flask (Nunc, Germany) and exposed to BDE-209 at 1 µmol L^−1^. The sampling for immunoblotting analyses were done after 7 and 15 days. At each time of sampling, two replicates were realized. After the treatment, the cells were recovered by trypsinization and centrifugation, as previously described, incubated for 30 min on ice in lysis buffer (1:4) (1% NP-40, 0.5% sodium deoxycholate, 0.1% sodium dodecyl sulfate (SDS), cocktail of protease inhibitors), and sonicated. Protein concentration was measured in total lysate, according to the method described by Lowry [45]. Experiments were carried out in duplicate.

#### 2.3.2. Immunoblotting

Equivalent amounts of proteins (20 μg) were loaded on pre-cast gel for SDS–polyacrylamide electrophoresis (SDS-PAGE), (Bio-Rad, Hercules, CA, USA) and blotted using a Trans Blot Turbo Transfer System (Bio-Rad, Hercules, CA, USA). The correct amount of protein loading was confirmed by red Ponceau staining. Filters were used for protein detection by primary antibodies (AbI) specifically for p53, extracellular signal-regulated kinase 1 (ERK1), phospho-AMP-activated protein kinase (AMPK), hypoxia-inducible factor (HIF), and nuclear factor (erythroid-derived 2)-like 2 (NRF2) (Sigma-Aldrich, Dorset, UK; Santa Cruz, CA, USA). In relation to the origin of the AbI, the appropriate secondary antibodies were used (anti-mouse or anti-rabbit, anti-goat secondary antibody conjugated with horseradish peroxidase) (GAR/M-HRP Bio-Rad, Hercules, CA, USA). The signals originated by immunoreaction were detected using enhanced chemo-luminescent (ECL) reagents (Bio-Rad). Images were obtained, photographed, and digitalized with Chemi Doc XRS (Bio-Rad, Hercules, CA, USA), and further analyzed with Image Lab software (Bio-Rad, Hercules, CA, USA). The results were expressed as fold increase of each treatment in relation to the control, representing the mean value of three separate experiments.

### 2.4. Statistical Analysis

Statistical differences among the groups were assessed by one-way ANOVA analyses, followed by the Bonferroni or Games Howell test, depending on the homogeneity of the variables. The normality of the variables was confirmed by the Shapiro–Wilk test, and homogeneity of variance by the Levene test. The significance level was 95% in all cases (*p* < 0.05). All the data were analyzed by the computer application SPSS for Windows^®^ (version 20.0, SPSS Inc., Chicago, IL, USA).

## 3. Results

### 3.1. Cytotoxicity Assay

**Figure 1:**
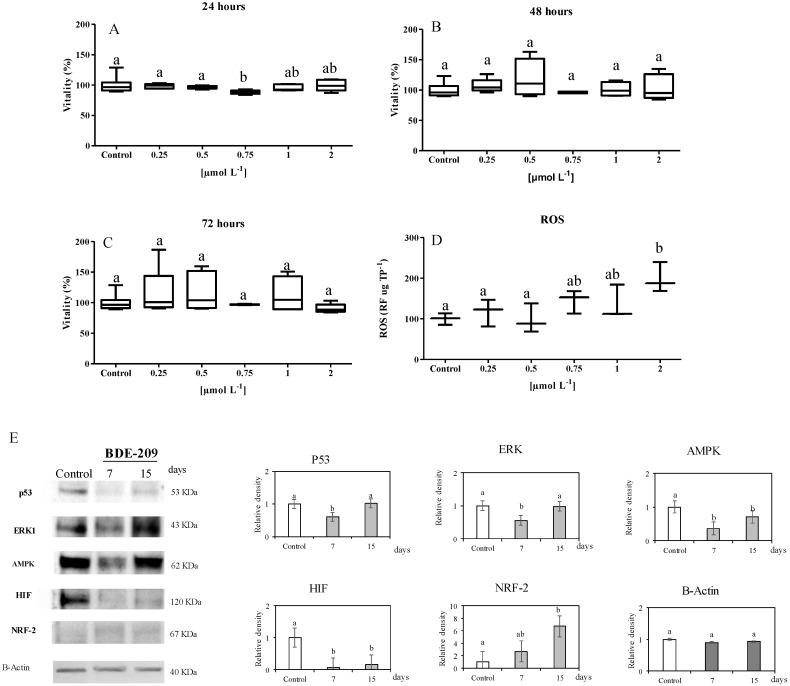
(**A**–**C**) Cytotoxicity of SAF-1 cells (fibroblast from *Sparus aurata*) exposed to different concentrations of polybrominated diphenyl ether-209 (BDE 209) (0.25–2 µmol L^−1^) for 24, 48, and 72 h. (**D**) Reactive oxygen species (ROS) production, expressed as relative fluorescence×µg total proteins^−1^, in SAF-1 cells exposed to different concentrations of BDE 209 (0.25–2 µmol L^−1^) for 72 h. (**E**) Immunoblotting of some proteins related to cell cycle (p53), proliferation (ERK), energetic balance (HIF, AMPK), and oxidative stress (NRF-2) in SAF-1 cells control: cell not exposed to the compound. Immunoblotting of actin protein is used as internal control for total proteins. Bars represent the mean ± SEM (n = 6). Statistically significant differences vs. the control (ANOVA; *p* ≤ 0.05) are denoted using different letters.

The effects of BDE 209 on the vitality of SAF-1 cells were investigated by MTTs. The results showed that only the dose 0.75 µmol L^−1^ of BDE 209 at 24 h significantly altered the cell vitality, with respect to the control samples (88.4 ± 1.4% of vitality; *p* = 0.03) (Figure 1A–C).

### 3.2. Evaluation of Intracellular ROS

A significant production of ROS was induced after 72 h of incubation, with respect to the control (*p* < 0.05), by the higher concentration of BDE 209 (2 µmol∙L^−1^) samples (Figure 1D).

### 3.3. Immunoblotting of Cell Cycle Biomarkers

Western blot analysis was carried out on SAF-1 cells in order to evaluate the presence and levels of selected proteins in response to the treatment, with a selected sub-lethal concentration of BDE-209 (1 µmol L^−1^) for a long time (7 and 15 days). The results are illustrated in Figure 1E. SAF-1 cells treated with BDE-209 showed a significant decrease (*p* < 0.05) in the levels of p53 at 7 days, but not at 15 days, with respect to the control. Regarding the marker of cell proliferation, levels of ERK1 significantly decreased (*p* < 0.05) with respect to the control at 7 days, but not at 15 days. The selected markers of energetic balance (AMPK and HIF) significantly decreased in cells treated with BDE-209 at 7 and 15 days (*p* < 0.05). Finally, the level of the marker related to the oxidative stress, NRF-2, significantly increased, with respect to the control (*p* < 0.05).

## 4. Discussion

The aim of the present study was to use the SAF-1 cell line as an in vitro model system to investigate the molecular mechanisms involved in cell cycle, cell metabolism, and oxidative stress produced by BDE-209, one of the most abundant PBDEs present in the environment and in wildlife [46,47,48].

Overall, PBDEs failed to affect SAF-1 cells after 24, 48, or 72 h of exposure. Although other polybrominated compounds—such as BDE-47 or BDE-99—have been shown to present toxicity in other models at the same or lower concentrations than used in our experiment [39], it has been demonstrated that low bromine BDE congeners (such as BDE-99 and 47) have been proved to be more toxic than higher bromine BDE congeners (as BDE-209) [49]. In fact, our results showed an increased trend in cell vitality with lower doses at 48 and 72 h, which agrees with previous research on different cell lines: in the ZFL cell line (liver from zebra fish), it was demonstrated that BDE-209 did not affect cell vitality, even at 96 h, at doses of up to 25 µmol L^−1^ [39]; on PC12 cells, concentrations ranging from 6.25 to 12.5 µmol L^−1^ (higher than the concentrations used in the present experiment) did not influence the vitality of cells [50]; also, on fibroblast cells obtained from *Stenella attenuate*, BDE-209 concentrations ranging from 260 nmol L^−1^ to 10 µmol L^−1^ did not determine a reduction in cell vitality [51].

Surprisingly, the vitality of SAF-1 cells was significantly decreased at 24 h with the 0.75 µmol∙L^−1^, while higher doses failed to decrease cell vitality in the same time. There has been little discussion about the fact that many endocrine-disrupting chemicals do not generate the standard trend of dose-response curves seen for other types of compounds [52], showing that lower concentrations of the contaminant could produce higher effects. In fact, it has been reported that bisphenol A [53], p-nonyphenol [54], and atrazine [55] produced this kind of response on different models. This could be a possible explanation for our vitality results.

Additionally, after PBDEs exposure, SAF-1 cells showed an increase of the intracellular ROS levels, in a dose-response manner (Figure 1D–F). Our results are in accord with other works on HepG2 [36,56] or in Neuro-2a [57] that reported that the PBDEs exposure induces oxidative stress. In addition to in vitro studies, an in vivo experiment showed that BDE-209 increased lipid peroxidation on germ cells from mice and decreased their antioxidant defenses [58].

However, the exact mechanisms by which these contaminants affect cell homeostasis, response to stress, metabolism, and cell cycle remain unclear. With the aim to elucidate this issue, the levels of different proteins related to the cell cycle, cell proliferation, energetic balance, and oxidative stress were analyzed on SAF-1 cells after exposure to a sub-lethal concentration of BDE-209 (1 µmol L^−1^) for 7 and 15 days.

After 7 and 15 days of BDE-209 exposure, p53 levels were significantly affected by PBDE treatment. p53 plays an important role in cell cycle regulation [59], increasing its levels in situations that can induce DNA damage, and assisting its reparation by promoting cell cycle arrest. For this reason, p53 is considered a biomarker relating to cell protection [60]. Surprisingly, BDE-209 produced a significant decrease of p53 levels after 7 days of treatment. This observation contrasts with other in vitro works that reported an increase of p53 expression in Neuro-2 cells exposed to BDE, BDE-47 and 99 [34], or in zebra fish embryos after exposure to a mix of PBDEs [61], as well as the increase of p53 expression in SH-SY5Y cells after BDE-47 treatment [62]. Nevertheless, the complexity of these pathways complicates the interpretation of these results, as is true that the decrease of p53 observed in our experiment could avoid DNA reparation and cell cycle arrest that p53 leads under stress conditions, increasing the susceptibility to mutagenesis and cell cycle disruption.

ERK1 plays a main role in cell activation, promoting cell proliferation and differentiation [63,64,65]. Our results revealed that ERK1 levels were significantly decreased at 7 days, which contrasts with other reports that showed PBDEs exposure significantly increased the levels of ERK1/2 in OVCAR-3 cells [66], cerebellar granule neurons [67], and HeLa cells [33]. However, the decrease of ERK1 levels could exacerbate the damage produced by BDE-209, as it has been suggested with HepG2 exposed to 1,2-dichloroethane [68]. Since ERK1/2 activation plays a protective role towards oxidative stress and others cell insults, its inhibition may result in a reduction of protective effects [69], however further research is needed to clarify this issue.

The enzymatic complex AMPK is activated by the increase of AMP/ATP ratio, and is therefore considered an indicator of cell energy levels [70]. Our results revealed that AMPK levels significantly decreased at both 7, and 15 days. In this sense, we hypothesized that a decrease of ATP levels could be due to a prolonged activity of antioxidant enzymes and phase I/II enzymes. The increase of intracellular ROS observed in our experiment seems to support our hypothesis. However, our results contrast with other reports that showed a decrease on ATP levels after BDE-47 or 99 exposure on isolated mitochondria from rat liver [71], correlating the PBDEs exposure with an increase of AMPK levels [70]. Nevertheless, a decrease of AMPK levels may be associated with changes in metabolism (low levels of ATP which leads anaerobic conditions), as described in cancer development [72] and in various types of cancer [73]. In fact, due to the fact that AMPK regulates energy levels, reinforces metabolic checkpoints, and inhibits cell growth [73], this protein is considered a tumor suppressor—therefore, the decrease of AMPK levels observed in our experiment could entail cancer promotion, although the mechanisms implicated in PBDEs effects are still unclear.

In our experiment, levels of HIF-1 showed a significant decrease after PBDEs exposure, which contrasts with some data reported by other authors that showed HIF-1 is increased under oxidative stress situations [74,75,76]. During a situation of oxidative stress, HIF-1 is normally activated [74,77], being translocated to the nucleus, and activates hypoxia-responsive elements (HRE) through the ARNT (aryl hydrocarbon receptor nuclear translocator). In this sense, it has been reported that HIF-1 activity could be influenced by the aryl hydrocarbon receptor (AhR) [78], which is activated by xenobiotics (such as polybrominated dibenzo-p-dioxins and dibenzofurans (PBDD/Fs)) and interacts with ARNT. Interestingly, some studies on the effects of PBDD/Fs on health suggested that this compound can produce a similar toxicity profile to its halogenated homologs [79], and that it can be hydroxylated under determinate conditions [80]. For this reason, AhR agonists can downregulate the HIF-1 pathway due to competition for ARNT [81,82,83]. This could be a plausible explanation of our results, although more research is needed to better understand this aspect.

NRF-2 plays protective roles, leading the expression of a wide range of antioxidants and phase II detoxification genes [84]. In our experiment, the levels of NRF-2 increased, suggesting that PBDEs exposure was able to activate the NRF-2 response in SAF-1 cells, probably via oxidative stress. Our observations agree with other works that reported the upregulation of NRF-2 via oxidative stress after PBDEs exposure, both in vivo [85] and in vitro [57,86].

## 5. Conclusions

Our results show that increased doses of PBDE-209 concentrations produced—in our model system—oxidative stress at short-term exposure. In addition, several pathways related to cell cycle, cell signaling, energy balance, and oxidative stress are influenced by long-term exposure to sub-lethal doses of the compound. Overall, our results suggest that the cellular response to low doses of PBDEs could be attenuated after long-term exposure by several mechanisms, such as ARNT competition or decreased energy levels. Some biomarkers indicate that exposure to PBDE-209 could decrease their protective effect, promoting cellular damage mainly through oxidative stress. This situation, together with anaerobic metabolism, could promote cellular transformation. Further studies are needed to ascertain the potential impact of different PBDEs on fish biology, and in this context, the use of in vitro models can represent a good alternative to understand the molecular mechanisms involved.

## Abbreviations

AhR	Aryl hydrocarbon receptor
AMPK	Adenosine 5′-monophosphate-activated protein kinase
AREs	Antioxidant response elements
ARNT	Aryl hydrocarbon receptor nuclear translocator
ERK-1	Extracellular signal–regulated kinase 1
HIF-1	Hypoxia inducible factor 1
NRF-2	Nuclear factor (erythroid-derived 2)-like 2
PBDEs	Polybrominated diphenyl ethers
PBDD/Fs	Polybrominated dibenzo-p-dioxins and dibenzofurans
ROS	Reactive oxygen species
